# Plastid genome of *Passiflora tripartita* var. *mollissima* (poro-poro) from Huánuco, Peru

**DOI:** 10.12688/f1000research.138150.1

**Published:** 2023-07-07

**Authors:** Flavio Aliaga, Mario Zapata-Cruz, Silvia Ana Valverde-Zavaleta

**Affiliations:** 1 Capítulo de Ingeniería Agronómica, Consejo Departamental de La Libertad (CDLL), Colegio de Ingenieros del Perú (CIP), Trujillo, 13008, Peru; 2 Dirección de Investigación, Innovación y Responsabilidad Social, Universidad Privada del Norte, Trujillo, 13011, Peru; 3 Plant Science Laboratory (PSL), Trujillo, 13009, Peru

**Keywords:** Plastid genome, Passifloraceae, Passiflora tripartita var. mollissima, poro-poro, native fruit, Huánuco, Peru

## Abstract

*Passiflora tripartita* var. *mollissima*, known locally as poro-poro, is an important native fruit used in traditional Peruvian medicine with relevant agro-industrial and pharmaceutical potential for its antioxidant capacity for human health. However, to date, only a few genetic data are available, which limits exploring its genetic diversity and developing new genetic studies for its improvement. We report the poro-poro plastid genome to expand the knowledge of its molecular markers, evolutionary studies, molecular pathways, and conservation genetics. Total genomic DNA was extracted from fresh leaves (herbarium voucher: USM:MHN331530). The DNA was sequenced using Illumina Novaseq 6000 platform providing 163,451 bp in length, with a large single-copy region of 85,525 bp and a small single-copy region of 13,518 bp, separated by a pair of inverted repeat regions (IR) of 32,204 bp, and the overall GC content was 36.87%. The chloroplast genome contains 129 genes (112 genes were unique and 17 genes were found duplicated in each IR region), including 85 protein-coding genes, 37 transfer RNA-coding genes, seven ribosomal RNA-coding genes, and 14 genes with introns (12 genes with one intron and two genes with two introns). The phylogenetic tree reconstructed based on single-copy orthologous genes and maximum likelihood analysis demonstrates poro-poro is most closely related to *Passiflora menispermifolia* and *Passiflora oerstedii.* In summary, our study provides the basis for developing new molecular markers that constitutes a valuable resource for studying molecular evolution and domestication. It also provides a powerful foundation for conservation genetics research and plant breeding programs. To our knowledge, this is the first report on the plastid genome of *Passiflora tripartita* var. *mollissima* from Peru.

## Introduction

*Passiflora tripartita* var. *mollissima* (Kunth) Holms-Niels. & P.M. Jørg ( ITIS, 2022) previously known as *Passiflora mollissima* (Kunth) Bailey ( Primot *et al.*, 2005), is a semi-perennial fruit plant ( Mayorga *et al.*, 2020). It is a diploid species with a small number of chromosomes (2n = 18) ( Coppens D’Eeckenbrugge, 2001), which is placed in the section Elkea of supersection Tacsonia of subgenus *Passiflora* belonging to the Passifloraceae family ( Segura *et al.*, 2005; Ocampo & Coppens d’Eeckenbrugge, 2017). Poro-poro is a native fruit of the Andean region ( Ocampo & Coppens d’Eeckenbrugge, 2017). It grows in the Peruvian highlands in the departments of Ancash, Junín, Moquegua, Huancavelica, and Huánuco at altitudes of 1,000–4,000 m.a.s.l. ( Tapia & Fries, 2007; Ríos-García, 2017). It is widely used in traditional medicine ( Ríos-García, 2017) and is considered one of the best *Passiflora* species based on its organoleptic characteristics ( Primot *et al.*, 2005). This fruit provides a source of vitamins (A, B3, and C) and minerals (magnesium, potassium, phosphorus, sodium, chlorine, iron, calcium, sulfur, zinc, copper, selenium, cobalt, and nickel) ( Leterme *et al.*, 2006; Chaparro-Rojas *et al.*, 2014). In addition, it has an elevated antioxidant activity and high content of carotenoids (118.8 mg β-carotene), phenols (460.1 mg gallic acid), and flavonoids (1907.6 mg catechin/100 g) ( Leterme *et al.*, 2006; Chaparro-Rojas *et al.*, 2014). Specifically, the high concentration of flavan-3-ols (a group of bioactive compounds) has been associated with beneficial effects on human health, such as cardiovascular protection, neurodegenerative diseases, and as an anti-cancer, anti-microbial, and anti-parasitic agent ( Giambanelli *et al.*, 2020; Luo *et al.*, 2022).

Plastome sequences of more than 800 sequenced genomes are small in size with high copy numbers and conserved sequences, enabling a significant understanding of plant molecular evolution, structural variations, and evolutionary relationships of plant diversity ( Daniell *et al.*, 2016; Dobrogojski *et al.*, 2020). The plastid genome has a quadripartite structure: a large single-copy (LSC) of 80–90 kilobase pairs (kb), a small single-copy (SSC) of 16–27 kb, and two sets of inverted repeats (IRa and IRb) of 20–28 kb, with 110–130 unique genes, including protein-coding genes, transfer RNA (tRNA), and ribosomal RNA (rRNA) ( Ozeki *et al.*, 1989; Wang & Lanfear, 2019). In recent years, declining genome sequencing costs resulted in more than 780 complete plant genomes of different species becoming available ( Marks *et al.*, 2021; Sun *et al.*, 2022). Recently, some *Passiflora* plastid genomes such as *Passiflora edulis* ( Cauz-Santos *et al.*, 2017), *Passiflora xishuangbannaensis* ( Hao & Wu, 2021), *Passiflora caerulea* ( Niu *et al.*, 2021), *Passiflora serrulata* ( Mou *et al.*, 2021), *Passiflora foetida* ( Hopley *et al.*, 2021), and *Passiflora arbelaezii* ( Shrestha *et al.*, 2019), became publicly available. However, despite the scarcity of genomic information on underutilized crops ( Gioppato *et al.*, 2019), we have only begun to investigate the genomics of plants of great importance for plant breeding programs. The aim of the present study was to sequence, assemble, and annotate the plastid genome of poro-poro to contribute to plant breeding programs. In the present study, we report the first plastid genome sequence submitted for an isolate of *Passiflora tripartita* var. *mollissima* from Peru, a species with great agro-industrial and pharmaceutical potential because of its beneficial characteristics for human health.

## Methods

### Plant materials

In November 2022, the fresh leaves of *Passiflora tripartita* var. *mollissima* were collected from Raccha Cedrón locality of Quisqui District, Huánuco Province from Peru (9°53′37″S, 76°26′02″W, altitude 2,945 m.a.s.l.). A herbarium voucher specimen (USM<PER>:MHN331530) was deposited in the Herbario San Marcos (USM) of the Museo de Historia Natural (MHN) at the Universidad Nacional Mayor de San Marcos (UNMSM) (see the *Extended data*, Aliaga *et al.*, 2023a).

### DNA extraction

Total genomic DNA was extracted from approximately 100 mg fresh leaves (from voucher number USM<PER>:MHN331530) according to Doyle’s (1991) method with slight modifications. The DNA isolation buffer consisted of buffer cetyl-trimethyl ammonium bromide (CTAB) 3% (30g/L CTAB, 100 mM Tris-HCl pH 8.0, 10nM EDTA, 1.4 M NaCl, 0,2% 2-mercaptoethanol), 70% ethanol, chloroform-isoamyl alcohol (24:1), 10 mM ammonium acetate, isopropanol, TE buffer (10 mM Tris-H, 1 mM EDTA), and RNAase A (10 ug/ml). Genomic DNA quality was assessed using a fluorometry-based Qubit (Thermo Fisher Scientific, USA, catalog number: Q33238) coupled to a Broad Range Assay kit (Thermo Fisher Scientific, USA, catalog number: Q33230). High-quality DNA (230/260 and 260/280 ratios >1.8) were normalized (20 ng/μL) to examine its integrity using 1% (w/v) agarose gel electrophoresis (see the *Extended data*, Aliaga *et al.*, 2023b) with the following equipment: Horizontal gel system (Fisher Scientific, Denmark, catalog number: 11833293, 150mm (length), 100 mm (width)), Transilluminator (Fisher Scientific, Spain, catalog number: 12864008), and digital camera (Canon, Spain, catalog number: 2955C002); Reagents: TAE buffer (40 mM Tris, 20mM NaAc, 1mM EDTA, pH 7.2), loading buffer 6X (Promega, USA, catalog number: G1881, 0.4% orange G, 0.03% bromophenol blue, 0.03% xylene cyanol FF, 15% Ficoll ^®^ 400, 10mM Tris-HCl pH 7.5 and 50mM EDTA pH 8.0) and Ethidium bromide (Promega, USA, catalog number H5041, 10 mg/ml), and 1 Kb Plus DNA Ladder (ThermoFisher, USA, catalog number: 10787018).

### Genome sequencing, assembly, and annotation

Qualified DNA was fragmented, and the TruSeq Nano DNA kit (Illumina, San Diego, CA, USA, catalog number: FC-121-4001) was used to construct an Illumina paired-end (PE) library. PE sequencing (2 × 150 bp) was performed using the Illumina NovaSeq 6000 platform ( Modi *et al.*, 2021) (Illumina, San Diego, Ca, USA, catalog number: 20012850) (Macrogen, Inc., Seoul, Republic of Korea). All adapters and low-quality reads were removed using the FastQC ( Wingett & Andrews, 2018) and Cutadapt ( Martin, 2011) programs. PE reads (2 × 150 bp) were evaluated for quality using QUAST ( Gurevich *et al.*, 2013) analysis, and subsequent steps used clean data. Then, clean reads obtained were assembled into a circular contig using NOVOPlasty v.4.3 ( Dierckxsens *et al.*, 2017), with *P. edulis* (NC_034285) as the reference ( Cauz-Santos *et al.*, 2017). The plastid genome was annotated using the Dual Organellar GenoMe Annotator GeSeq ( Tillich *et al.*, 2017) and CpGAVAS2 ( Shi *et al.*, 2019). A circular genome map was constructed using OGDRAW v.1.3.1 ( Greiner *et al.*, 2019). Finally, the completed sequences were submitted to the NCBI GenBank under the accession number OQ910395 ( GenBank, 2023).

### Phylogenetic analysis

We used 26 complete plastome sequences to infer the phylogenetic relationships among Passiflora species, and *Vitis vinifera* was used as an outgroup (see the *Extended data*, Aliaga *et al.*, 2023c). Single-copy orthologous genes were identified using the Orthofinder version 2.2.6 pipeline ( Emms & Kelly, 2019). For each gene family, the nucleotide sequences were aligned using the L-INS-i algorithm in MAFFT v7.453 ( Katoh & Standley, 2013). A phylogenetic tree based on maximum likelihood (ML) was constructed using RAxML v8.2.12 ( Stamatakis, 2014) with the GTRCAT model. A phylogenetic ML tree was reconstructed and edited using MEGA 11 ( Tamura *et al.*, 2021) with 1000 replicates.

## Results

### Plastome of *Passiflora tripartiva* var. *mollissima*

**Figure 1.**
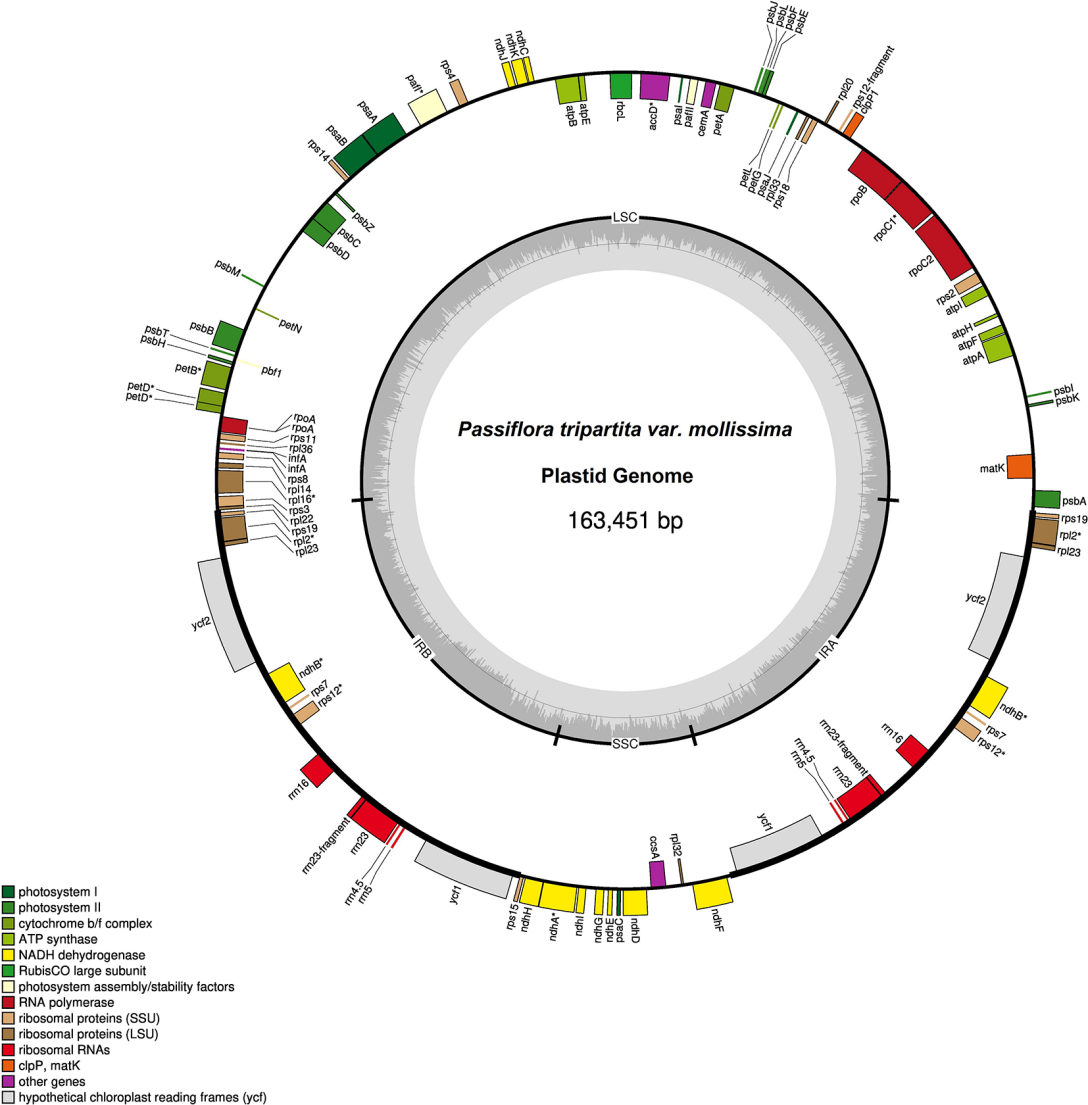
Plastid genome of *Passiflora tripartita* var. *mollissima.* The thick lines indicate the IR1 and IR2 regions, which separate the large single-copy (LSC) and small single-copy (SSC) regions. Genes marked inside the circle are transcribed clockwise, and genes marked outside the circle are transcribed counterclockwise. Genes are color-coded based on their function, shown at the bottom left. The inner circle indicates the inverted boundaries and guanine and cytosine (GC) content.

The plastid genome sequences of *P. tripartita* var. *mollissima* (poro-poro) ( Figure 1) was 163,451 bp in length, with a typical quadripartite structure consisting of a large single-copy (LSC) region of 85,525 bp (52.32% in total) and a small single-copy (SSC) region of 13,518 bp (8.27%), separated by a pair of inverted repeat regions (IRs) of 32,204 bp (19.70%). The poro-poro plastome is 12,045 bp longer than that of one of the most economically important species, passion fruit ( *P. edulis*) ( Cauz-Santos *et al.*, 2017), and is only 7,117 bp longer than that of the longest *Passiflora* plastome reported, i.e., *P.* *arbelaezii* ( Shrestha *et al.*, 2019). The plastome sequence of poro-poro has a *similar* quadripartite architecture to other plants ( Ohyama *et al.*, 1986; Shinozaki *et al.*, 1986; Nguyen *et al.*, 2021). However, the LSC region is 4,150 bp longer than that of *P. xishuangbannaensis* but is 98bp, 195 bp, and 1,927 bp shorter than that of *P. caerulea*, *P. edulis*, and *P. arbelaezii*, respectivety. The SSC region is 121 bp, 140 bp, 359 bp, and 754 bp longer than that of *P. caerulea*, *P. edulis*, *P. xishuangbannaensis*, and *P. arbelaezii*, respectively. The IRs regions are 6,024 bp, 6,050 bp, and 11,600 longer than that of *P. caerulea*, *P. edulis*, and *P. xishuangbannaensis*, respectively; however, it is 2,972 bp shorter than that of *P. arbelaezii* ( Cauz-Santos *et al.*, 2017; Shrestha *et al.*, 2019; Hao & Wu, 2021; Niu *et al.*, 2021). The plastome structure of the *P. tripartita* var. *mollissima* consisted of A = 30.79%, T(U) = 32.34%, C = 18.67% and G = 18.20%. The overall AT content of the plastid genome was 63.13%, whereas the overall GC content was 36.87% as similar to that of other reported chloroplast genomes from the same family, such as 36.90% in *P. arbelaezii* ( Shrestha *et al.*, 2019), 37% in *P. edulis* and *P. serrulata* ( Cauz-Santos *et al.*, 2017; Mou *et al.*, 2021), 37.03% in *P. caerulea* ( Niu *et al.*, 2021), and 37.1% in *P. xishuangbannaensis* ( Hao & Wu, 2021).

Poro-poro plastid genome annotation identified 129 genes, of which 112 were unique, and 17 were duplicated in the inverted repeat (IR) region. The plastome contained 85 protein-coding genes, 37 transfer RNA (tRNA)-coding genes, seven ribosomal RNA (rRNA)-coding genes, and 14 genes with introns (12 genes with one intron and two genes with two introns), as shown in Table 1. The poro-poro plastid genome contained 112 unique genes, of which there were 29 tRNA genes, four rRNA genes, and 79 protein-coding genes. The latter comprised 20 ribosomal subunit genes (nine large subunits and 11 small subunit), four DNA-directed RNA polymerase genes, 46 genes were involved in photosynthesis (11 encoded subunits of the NADH oxidoreductase, seven for photosystem I, 15 for photosystem II, six for the cytochrome b6/f complex, six for different subunits of ATP synthase, and one for the large chain of ribulose biphosphate carboxylase), eight genes were involved in different functions, and one gene was of unknown function ( Table 2).

**Table 1.** Plastid genome features of the *P. tripartita* var. *mollissima.*

Features	Poro-poro ^1^
Genome size (bp)	163,451
^a^LSC length (bp)	85,525
^b^SSC length (bp)	13,518
^c^IR length (bp)	32,204
Total GC content (%)	36.87
^d^A content (%)	30.79
^e^T(U) content (%)	32.34
^f^G content (%)	18.20
^g^C content (%)	18.67
Total number of genes	129
Protein-coding genes	85
^h^rRNA coding genes	7
^i^tRNA coding genes	37
Genes duplicated in IR regions	17
Total introns	14
Single introns (gene)	12
Double introns (gene)	2

^1^ Poro-poro is the common name of *Passiflora tripartita* var. *mollissima* in Peru.

^a^ LSC: a large single-copy.

^b^ SSC: a small single-copy.

^c^ IR: inverted repeat.

^d^ A: adenine.

^e^ T(U): thymine (uracil).

^f^ G: guanine.

^g^ C: cytosine.

^h^ rRNA: ribosomal RNA.

^i^ tRNA: transfer RNA.

**Table 2.** Genes present in the plastid genome of *P. tripartita* var. *mollissima.*

Group of genes	Gene names
Photosystem I	psaA, psaB, psaC, psaI, psaJ, ycf3 **, ycf4
Photosystem II	psbA, psbB, psbC, psbD, psbE, psbF, psbH, psbI, psbJ, psbK, psbL, psbM, psbN, psbT, psbZ
Cytochrome b/f complex	petA, petB, petD *, petG, petL, petN
ATP synthase	atpA, atpB, atpE, atpF, atpH, atpI
NADH dehydrogenase	ndhA *, ndhB * (X2), ndhC, ndhD, ndhE, ndhF, ndhG, ndhH, ndhI, ndhJ, ndhK
RubisCO large subunit	rbcL
DNA-dependent RNA polymerase	rpoA, rpoB, rpoC1 *, rpoC2
Ribosomal proteins (SSU)	rps2, rps3, rps4, rps8, rps11, rps12 ** (X2), rps14, rps15, rps16, rps18, rps19 (X2)
Ribosomal proteins (LSU)	rpl2 * (X2), rpl14, rpl16 *, rpl20, rpl22, rpl23 (X2), rpl32, rpl33, rpl36
Acetyl-CoA carboxylase	accD
C-type cytochrome synthesis	ccsA
Envelope membrane protein	cemA
Protease	clpP
Translational initiation factor IF-1	infA
Maturase	matK
Component of TIC complex	yct1, ycf2
Unknown function protein-coding	ycf15 (X2)
Ribosomal RNAs	rrn4.5, rrn5 (X2), rrn16 (X2), rrn23 (X2)
Transfer RNAs	trnA-UGC * (X2), trnC-GCA, trnD-GUC, trnE-UUC, trnF-GAA, trnG-GCC, trnG-UCC *, trnH-GUG, trnI-CAU (X2), trnI-GAU * (X2), trnK-UUU *, trnL-CAA (X2), trnL-UAA *, trnL-UAG, trnM-CAU (X2), trnN-GUU (X2), trnP-UGG, trnQ-UUG, trnR-ACG (X2), trnR-UCU, trnS-GCU, trnS-GGA, trnS-UGA, trnT-GGU, trnT-UGU, trnV-GAC (X2), trnV-UAC *, trnW-CCA, trnY-GUA

* Gene contains one intron.

** gene contains two introns; (X2) indicates two gene copies in IRs.

In the plastid genome, 14 genes contained introns distributed as follows: the LSC, SSC, and IRs regions contained eight genes (petD, rpl16, rpoC1, trnG-UCC, trnK-UUU, trnL-UAA, trnV-UAC, and ycf3), one gene (ndhA), and five genes (ndhB, rpl2, rps12, trnA-UGC, and trnI-GAU) respectively. Similarly, these genes included six protein-coding genes, each with a single intron (petD, ndhA, ndhB, rpoC1, rpl2, and rpl16); six tRNA genes, each with a single intron (trnA-UGC, trnG-UCC, trnI-GAU, trnK-UUU, trnL-UAA, and trnV-UAC); and two protein-coding genes with two introns (ycf3 and rps12). Except for 17 genes that were duplicated in the IR region (ndhB, rps19, rpl2, rpl23, rps12, ycf15, rrn5, rrn16, rrn23, trnA-UGC, trnI-CAU, trnI-GAU, trnL-CAA, trnM-CAU, trnN-GUU, trnR-ACG, and trnV-GAC) all genes contained a single copy, as shown in Table 2. The plastome of *P. tripartita* var. *mollissima* contained eight genes (ycf1, ycf2, ycf15, rps16, rpl20, rpl22, accD, infA) that were lost or non-functional genes in *P. edulis*; and compared to *P. edulis*, it has one absent gene (trnfM-CAU), as previously reported ( Cauz-Santos *et al.*, 2017). In this study, the ycf1 sequence encodes a protein essential for plant viability and a vital component of the translocon on the inner chloroplast membrane (TIC) complex ( Kikuchi *et al.*, 2013), and ycf2 is a component of the ATPase motor protein associated with the TIC complex ( Kikuchi *et al.*, 2018).

### Phylogenetic reconstruction

To identify the evolutionary position of *Passiflora tripartita* var. *mollissima* in the Passifloraceae family, phylogenetic relationships based on the OrthoFinder clustering method were used to avoid erroneous rearrangements in phylogenetic tree reconstruction and provides a more reliable evolutionary analysis ( Gabaldón, 2005; Zhang *et al.*, 2012). The phylogenetic tree was constructed based on single-copy orthologous genes ( Emms & Kelly, 2019) and maximum likelihood analysis with the complete annotated protein sequences of 27 plastid genomes, of which 26 were from *Passiflora* species. One species, *Vitis vinifera*, was chosen as the outgroup.

**Figure 2.**
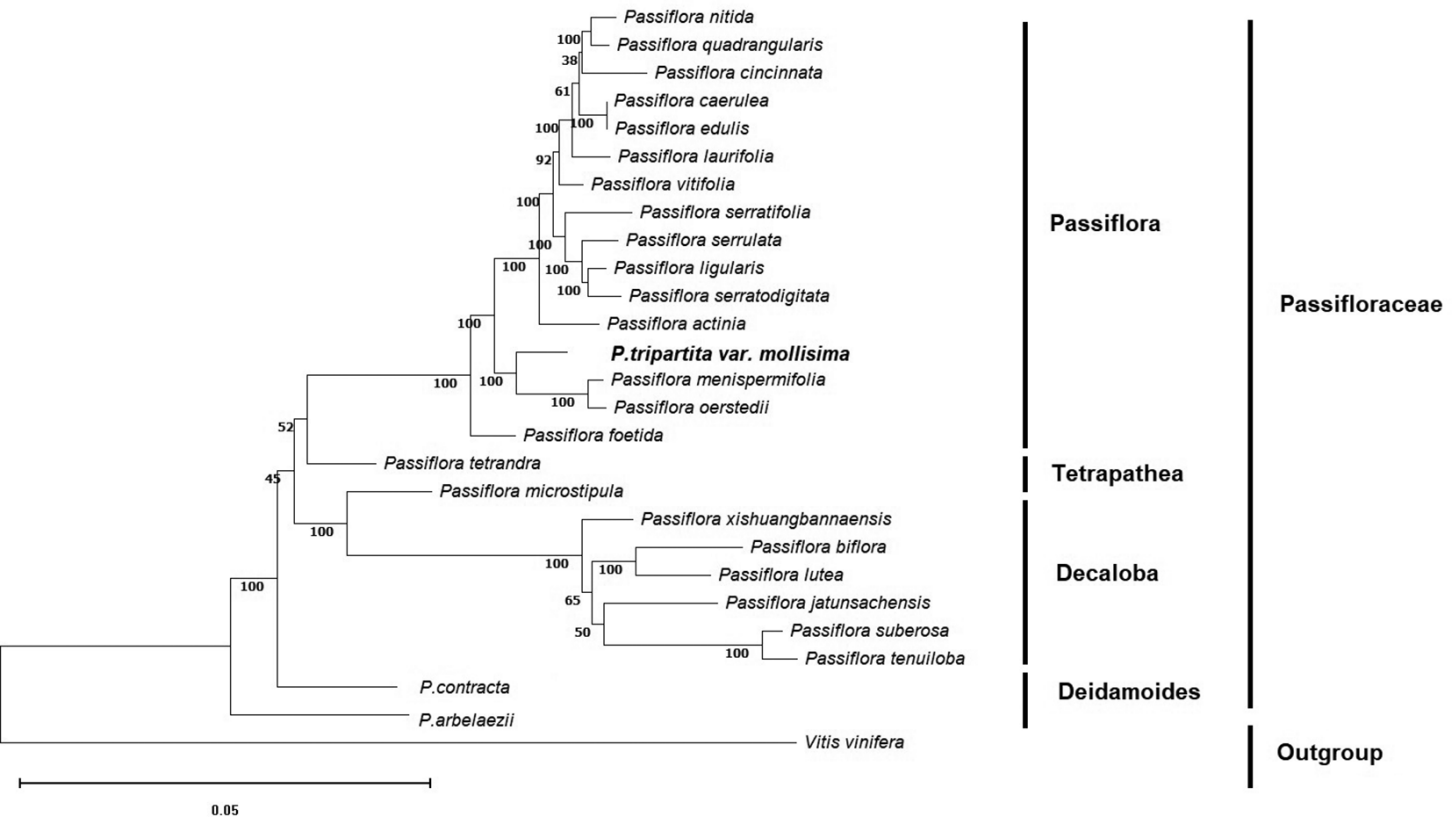
Phylogenetic tree of 27 plastid genomes using maximum likelihood analysis based on single-copy orthologous protein. Bootstrap values on the branches were calculated from 1000 replicates.

Maximum likelihood (ML) bootstrap values ranged from 38%–92% for seven of the 25 nodes. All nodes except the indicated ones (seven nodes) exhibited bootstrap support (BS) values of 100%. These *Passiflora* species were divided into four groups: subgenus *Passiflora* ( *P. nitida*, *P. quadrangularis*, *P. cincinnata*, *P. caerulea*, *P. edulis*, *P. laurifolia*, *P. vitifolia*, *P. serratifolia*, *P. serrulata*, *P. ligularis*, *P. serratodigitata*, *P. actinia*, *P. menispermifolia* and *P. oerstedii*), subgenus *Tetrapathea* ( *P. tetrandra*), subgenus *Decaloba* ( *P. microstipula*, *P. xishuangbannaensis*, *P. biflora*, *P. lutea*, *P. jatunsachensis*, *P. suberosa* and *P. tenuiloba*), and subgenus *Deidamoides* ( *P. contracta* and *P. arbelaezii*). The relationships between the four subgenera of *Passiflora* species ( *Passiflora, Tetrapathea, Decaloba*, and *Deidamoides*) were congruent and strongly supported by the same patterns as previously reported ( Cauz-Santos *et al.*, 2020; Pacheco *et al.*, 2020). These results resolved *Passiflora tripartita* var. *mollissima* belonging to the subgenus *Passiflora*, which was closely related to *P. menispermifolia* and *P. oerstedii* with 100% BS, and was sister to *P. tetrandra* (subgenus *Tetrapathea*), *P. biflora* (subgenus *Decaloba*), and *P. contracta* (subgenus *Deidamoides*), as shown in the cladogram ( Figure 2).

## Data Availability

Nucleotide: *Passiflora tripartita* var. *mollissima* chloroplast, complete genome. Accession number: OQ910395. https://identifiers.org/nucleotide:OQ910395 ( GenBank, 2023). Figshare: Herbarium specimen voucher of *Passiflora tripartita* var. *mollissima* (Kunth) Holms-Niels. & P.M. Jørg (USM:MHN331530). https://doi.org/10.6084/m9.figshare.23556654 ( Aliaga *et al*., 2023a). Figshare: Gel imagen of DNA isolate from poro-poro sample. https://doi.org/10.6084/m9.figshare.23560755 ( Aliaga *et al*., 2023b). Figshare: Details of the plastid genome sequences used for phylogenetic analysis. https://doi.org/10.6084/m9.figshare.23556834 ( Aliaga *et al*., 2023c). Data are available under the terms of the Creative Commons Attribution 4.0 International license (CC-BY 4.0).
