# Supplementary material for: Plastid genome of Passiflora tripartita var. mollissima (poro-poro) from Huánuco, Peru
Source: F1000Res. 2024 Feb 19;12:795. Originally published 2023 Jul 7. [Version 3] doi: 10.12688/f1000research.138150.3 (PMC10904978; doi:10.12688/f1000research.138150.3)
Supplement: Peer review history

# Reviewer report: Reviewer response for version 3

**DOI:** 10.5256/f1000research.162741.r248377

**Published:** 2024-02-22

**Authors:** Rahul G Shelke

**Affiliations:** 1 Independent Researcher, Amravati, India

Since the authors attentively considered every recommendation, I have nothing else to add.

Are the datasets clearly presented in a usable and accessible format, and the assembly and annotation available in an appropriate subject-specific repository?

Yes

Are sufficient details of the sequencing and extraction, software used, and materials provided to allow replication by others?

Yes

Are the rationale for sequencing the genome and the species significance clearly described?

Yes

Are the protocols appropriate and is the work technically sound?

Yes

Reviewer Expertise:

Organelle genome sequencing, Transcriptome assembling, Genetic diversity, Barcoding and DNA markers  etc

I confirm that I have read this submission and believe that I have an appropriate level of expertise to confirm that it is of an acceptable scientific standard.

# Reviewer report: Reviewer response for version 2

**DOI:** 10.5256/f1000research.160734.r236402

**Published:** 2024-02-14

**Authors:** Rahul G Shelke

**Affiliations:** 1 Independent Researcher, Amravati, India

I have thoroughly reviewed the manuscript and would like to bring to your attention a minor issue in Fig. 1.

 Upon careful examination, I have observed that certain genes, including rrn23, rrn23-fragment, infA, rpoA, etc., bear double annotations in the figure. I appreciate the authors' efforts in incorporating the suggested changes throughout the manuscript. However, I recommend addressing this specific matter to enhance clarity for readers.

 To rectify this, I propose that the extra annotations be removed from the GenBank file before generating the diagram. This correction will streamline the information presented in Fig. 1, ensuring a more concise and comprehensible representation for new readers.

 I believe implementing this adjustment will further enhance the overall quality of the manuscript.

Are the datasets clearly presented in a usable and accessible format, and the assembly and annotation available in an appropriate subject-specific repository?

Yes

Are sufficient details of the sequencing and extraction, software used, and materials provided to allow replication by others?

Yes

Are the rationale for sequencing the genome and the species significance clearly described?

Yes

Are the protocols appropriate and is the work technically sound?

Yes

Reviewer Expertise:

Organelle genome sequencing, Transcriptome assembling, Genetic diversity, Barcoding and DNA markers  etc

I confirm that I have read this submission and believe that I have an appropriate level of expertise to confirm that it is of an acceptable scientific standard.

# Reviewer report: Reviewer response for version 2

**DOI:** 10.5256/f1000research.160734.r236401

**Published:** 2024-01-19

**Authors:** Abdullah Abdullah

**Affiliations:** 1 Alpha Genomics Private Limited, Islamabad, Quaid-i-Azam University, Islamabad, Islamabad Capital Territory, Pakistan

The article can be accepted.

Are the datasets clearly presented in a usable and accessible format, and the assembly and annotation available in an appropriate subject-specific repository?

Yes

Are sufficient details of the sequencing and extraction, software used, and materials provided to allow replication by others?

Partly

Are the rationale for sequencing the genome and the species significance clearly described?

Partly

Are the protocols appropriate and is the work technically sound?

Partly

Reviewer Expertise:

I can assess all aspects of the manuscript and published up to 18 articles in the same field.

I confirm that I have read this submission and believe that I have an appropriate level of expertise to confirm that it is of an acceptable scientific standard.

# Reviewer report: Reviewer response for version 1

**DOI:** 10.5256/f1000research.151329.r191281

**Published:** 2023-09-04

**Authors:** Abdullah Abdullah

**Affiliations:** 1 Alpha Genomics Private Limited, Islamabad, Quaid-i-Azam University, Islamabad, Islamabad Capital Territory, Pakistan

Authors need to describe how the current genome will be helpful. I found many species of the genus *Passiflora *in the NCBI. So, authors need clear points about the need for the existing genome. In addition, all the genomes have similar features so the current genome is helpful for the study of evolution and phylogenetics.  

 The authors mentioned the number of reported genomes up to 800. This statement is not correct and is based on earlier observations. Now the number has increased significantly. 

 I found authors are not entirely aware of the utilization of some software. For example, authors mention fastqc for trimming while fastqc checks the quality of reads.

 The authors used many programs for annotations. I will suggest authors use GeSeq along with Chole, Aragorn and tRNAScan on GeSeq servers for annotation of transfer RNA. 

 The authors need to give some information on how the current genome is similar to or differs from other reported genomes of *Passiflora*.

Are the datasets clearly presented in a usable and accessible format, and the assembly and annotation available in an appropriate subject-specific repository?

Yes

Are sufficient details of the sequencing and extraction, software used, and materials provided to allow replication by others?

Partly

Are the rationale for sequencing the genome and the species significance clearly described?

Partly

Are the protocols appropriate and is the work technically sound?

Partly

Reviewer Expertise:

I can assess all aspects of the manuscript and published up to 18 articles in the same field.

I confirm that I have read this submission and believe that I have an appropriate level of expertise to confirm that it is of an acceptable scientific standard, however I have significant reservations, as outlined above.

# Response

**Published:** 2023-12-28

**Authors:** Flavio Aliaga

**Affiliations:** Universidad Privada del Norte, Trujillo, La Libertad, Peru

Corresponding Author: Flavio Aliaga

 Grupo de Investigación en Ecología Evolutiva, Protección de Cultivos, Remediación Ambiental, y Biotecnología (EPROBIO), Universidad Privada del Norte, Trujillo, Peru. 

 Dirección de Investigación, Innovación y Responsabilidad Social, Universidad Privada del Norte, Trujillo, 13011, Peru

 1. Authors need to describe how the current genome will be helpful. I found many species of the genus Passiflora in the NCBI. So, authors need clear points about the need for the existing genome.

**Answer**: We would like to thank reviewer 2 for the insightful response. We have considered your comment because the poro-poro plastid genome constitutes a valuable resource for studying the molecular evolution, phylogenetics, and domestication of species with beneficial characteristics for human health.

 2. In addition, all the genomes have similar features so the current genome is helpful for the study of evolution and phylogenetics. 

**Answer**: We are grateful to reviewer 2 for the constructive and valuable comments. Thank you.

 3. The authors mentioned the number of reported genomes up to 800. This statement is not correct and is based on earlier observations. Now the number has increased significantly.

**Answer**. We agree with the reviewer. The manuscript has been corrected:

-  " Plastome sequences from over 4000 species…”.

4. I found authors are not entirely aware of the utilization of some software. For example, authors mention fastqc for trimming while fastqc checks the quality of reads.

**Answer**. It has been revised, thank you.

 5. The authors used many programs for annotations. I will suggest authors use GeSeq along with Chole, Aragorn and tRNAScan on GeSeq servers for annotation of transfer RNA.

**Answer**. It has been revised, thank you.

 6. The authors need to give some information on how the current genome is similar to or differs from other reported genomes of Passiflora

**Answer**. Thank you for your comment. We have added the figure 2 titled “Comparison of IR/SC boundary regions of four *Passiflora* species”.

# Reviewer report: Reviewer response for version 1

**DOI:** 10.5256/f1000research.151329.r191279

**Published:** 2023-08-30

**Authors:** Rahul G Shelke

**Affiliations:** 1 Independent Researcher, Amravati, India

I extend my appreciation to the authors for their commendable efforts in extensively exploring the plastid genome of *Passiflora tripartita* var. *mollissima*, demonstrating remarkable depth and breadth in their approach. The manuscript substantially enhances our comprehension of this species, emphasizing its plastid genome, genetic diversity, and evolutionary relationships. This newfound insight carries far-reaching implications, fostering the conservation and sustainable utilization of this species. Consequently, the manuscript's significance is markedly elevated, making a noteworthy contribution to the scientific community's understanding of genetic resource stewardship.

 Here is a concise summary of the study's key points:

1. Implications and Novelty: The findings of the study hold significant implications. The genetic insights uncovered have the potential to catalyze further investigations in areas such as molecular evolution, conservation genetics, and the development of plant breeding programs. Notably, the manuscript's distinction as the first documentation of the plastid genome of *Passiflora tripartita* var. *mollissima *from Peru underscores its novel contribution to the scientific community.
2. Methodological Rigor: The authors' selection of the Illumina Novaseq 6000 platform for DNA sequencing is well-suited to the research objectives. The subsequent analyses and methodologies applied exhibit robustness and alignment with the research goals.

**Major comments: **

1. While the genes in Table 2 have been organized into categories, it is apparent that certain instances lack gene information or are unclear in their presentation. This calls for a restructuring of the table to ensure a heightened level of clarity and coherence.
2. In Table 2, the authors have indicated the absence of a duplicated copy of the rrn4.5 gene. It is advised that the authors review the annotation of this gene. Typically, rRNA genes are located within the IR regions and therefore exist in duplicated copies. However, in Figure 1 of the chloroplast genome, the rrn4.5 gene is depicted in a duplicated copy. I recommend that the authors rectify any discrepancies pertaining to the rrn4.5 gene both in the table and within the text.
3. The authors have provided comprehensive insights into the expansion and contraction of the LSC, SSC, and IR regions in the compared species. To further enhance reader comprehension, I recommend that the authors consider incorporating the corresponding IRSCOPE figure within the manuscript. This addition would allow readers to access detailed information more effectively and contribute to a clearer understanding of the discussed concepts.
4. Kindly provide information regarding the genes that are missing or subject to gene loss within the compared species.
5. In the abstract, the authors have included the statement: “In summary, our study provides the basis for developing new molecular markers that constitutes a valuable resource for studying molecular evolution and domestication.” However, upon reviewing the manuscript, it is evident that the authors have not conducted any SSR, tandem repeat, or DNA diversity analysis, nor have they proposed any markers. Given this situation, I recommend that the authors consider removing the phrase “developing new molecular markers” from the abstract to accurately reflect the scope of their study.

**Minor comments:**

1. The inclusion of specific methodology details such as "Total genomic DNA was extracted from fresh leaves (herbarium voucher: USM:MHN331531)" and "The DNA was sequenced using Illumina Novaseq 6000 platform" in the abstract might be better suited for the methodology section rather than the abstract itself. Focusing the abstract on the core findings and implications of the study could enhance its conciseness and clarity.
2. Add the full botanical name of *Passiflora tripartita* in the phylogeny tree to ensure consistency.
3. Gene names should be in italics.
4. It is recommended to include the coverage of the genome assembly within the main text of the manuscript.

The authors adeptly tackle gaps in our understanding of this species' genetics, comparative genomics, and evolutionary relationships. By thoughtfully engaging with reviewer feedback and subsequently refining their manuscript, the authors stand poised to elevate the impact and significance of their research within the realm of plant genomics and beyond. This process will significantly enhance the accuracy and reliability of gene classification and annotation, thereby elevating the overall quality of the manuscript.

Are the datasets clearly presented in a usable and accessible format, and the assembly and annotation available in an appropriate subject-specific repository?

Yes

Are sufficient details of the sequencing and extraction, software used, and materials provided to allow replication by others?

Yes

Are the rationale for sequencing the genome and the species significance clearly described?

Yes

Are the protocols appropriate and is the work technically sound?

Yes

Reviewer Expertise:

Organelle genome sequencing, Transcriptome assembling, Genetic diversity, Barcoding and DNA markers  etc

I confirm that I have read this submission and believe that I have an appropriate level of expertise to confirm that it is of an acceptable scientific standard, however I have significant reservations, as outlined above.

# Response

**Published:** 2023-12-28

**Authors:** Flavio Aliaga

**Affiliations:** Universidad Privada del Norte, Trujillo, La Libertad, Peru

Corresponding Author: Flavio Aliaga

 Grupo de Investigación en Ecología Evolutiva, Protección de Cultivos, Remediación Ambiental, y Biotecnología (EPROBIO), Universidad Privada del Norte, Trujillo, Peru. 

 Dirección de Investigación, Innovación y Responsabilidad Social, Universidad Privada del Norte, Trujillo, 13011, Peru

 The authors thank Dr. Rahul G. Shelke for taking the time and effort necessary to review the manuscript. We sincerely appreciate all the valuable comments and suggestions that helped us improve the quality of the manuscript.

**Answers to Reviewer #1**

Major comments:

 1. While the genes in Table 2 have been organized into categories, it is apparent that certain instances lack gene information or are unclear in their presentation. This calls for a restructuring of the table to ensure a heightened level of clarity and coherence.****

**Answer**: We agree with the reviewer. The manuscript has been revised, thanks.

 2. In Table 2, the authors have indicated the absence of a duplicated copy of the rrn4.5 gene. It is advised that the authors review the annotation of this gene. Typically, rRNA genes are located within the IR regions and therefore exist in duplicated copies. However, in Fiure 1 of the chloroplast genome, the rrn4.5 gene is depicted in a duplicated copy. I recommend that the authors rectify any discrepancies pertaining to the rrn4.5 gene both in the table and within the text.

**Answer**: We would like to thank the reviewer for highlighting this important comment. In fact, we checked and updated the duplicated copy of the rrn4.5 gene. Thank you.

 3. The authors have provided comprehensive insights into the expansion and contraction of the LSC, SSC, and IR regions in the compared species. To further enhance reader comprehension, I recommend that the authors consider incorporating the corresponding IRSCOPE figure within the manuscript. This addition would allow readers to access detailed information more effectively and contribute to a clearer understanding of the discussed concepts.

**Answer**: Thank you for your comment.  According to your suggestions, we have added the figure 2 titled “Comparison of IR/SC boundary regions of four *Passiflora* species”.

 4. Kindly provide information regarding the genes that are missing or subject to gene loss within the compared species.

**Answer**: Thank you for your reminding. Done.

 5. In the abstract, the authors have included the statement: “In summary, our study provides the basis for developing new molecular markers that constitutes a valuable resource for studying molecular evolution and domestication.” However, upon reviewing the manuscript, it is evident that the authors have not conducted any SSR, tandem repeat, or DNA diversity analysis, nor have they proposed any markers. Given this situation, I recommend that the authors consider removing the phrase “developing new molecular markers” from the abstract to accurately reflect the scope of their study.

**Answer**. We agree with the reviewer. The manuscript has been corrected:

- “In summary, our study constitutes a valuable resource for studying molecular evolution, phylogenetics, and domestication…”

Minor comments:

 1. The inclusion of specific methodology details such as "Total genomic DNA was extracted from fresh leaves (herbarium voucher: USM:MHN331531)" and "The DNA was sequenced using Illumina Novaseq 6000 platform" in the abstract might be better suited for the methodology section rather than the abstract itself. Focusing the abstract on the core findings and implications of the study could enhance its conciseness and clarity.

**Answer**. Yes, we agree with the reviewer. Done.

 2. Add the full botanical name of Passiflora tripartita in the phylogeny tree to ensure consistency.

**Answer**. Done.

 3. Gene names should be in italics. It is recommended to include the coverage of the genome assembly within the main text of the manuscript.

**Answer**. Done.

 4. The authors adeptly tackle gaps in our understanding of this species' genetics, comparative genomics, and evolutionary relationships. By thoughtfully engaging with reviewer feedback and subsequently refining their manuscript, the authors stand poised to elevate the impact and significance of their research within the realm of plant genomics and beyond. This process will significantly enhance the accuracy and reliability of gene classification and annotation, thereby elevating the overall quality of the manuscript.

**Answer**: We thank reviewer 1 for all constructive comments that led to substantial improvement of the manuscript. Thank you; it is much appreciated.
